# The Role of Neighborhood Social Context in the Link Between Cumulative Social Isolation and Cognitive Decline

**DOI:** 10.1093/geroni/igaf122.4270

**Published:** 2025-12-31

**Authors:** Anna Chupak, Yanping Jiang

**Affiliations:** University of South Carolina, Columbia, South Carolina, United States; Rutgers, The State University of New Jersey, New Brunswick, New Jersey, United States

## Abstract

Social isolation is prevalent among U.S. adults aged 50–80 and is associated with poor cognition and elevated dementia risk. Few studies have examined how variations in type and repeated exposure to social isolation, including differences by neighborhood context, influence cognitive trajectories. This study investigates how cumulative, subjective and objective social isolation impact adults’ cognitive function over time, including variations by neighborhood social cohesion and physical disorder. We used data from the Health and Retirement Study (2006–2016) on American adults aged 50–104 (N = 7,410). Objective and subjective social isolation were measured by the 5-item Steptoe Index and the 3-item UCLA Loneliness Scale, respectively. Cognitive function was assessed using combined memory and executive function scores. We applied weighted mixed-effects regressions to examine associations between each type of cumulative social isolation and cognitive decline, adjusting for sociodemographic and health covariates. Then, we stratified each association by perceived neighborhood social cohesion (e.g., trust) and physical disorder (e.g., crime). Compared to no social isolation, individuals with persistent objective social isolation experienced the steepest cognitive decline over 8 years (p = 0.02, SE = 0.02); this association was non-significant among those living in neighborhoods with low levels of physical disorder. Individuals with persistent subjective social isolation had worse baseline cognition (p < 0.01, SE = 0.18), but showed more gradual cognitive decline over time (p < 0.01, SE = 0.02). These findings highlight the need for community-level strategies alongside individual interventions to reduce loneliness and promote social connectedness and cognitive resilience in older adults. Improving neighborhood conditions may buffer the cognitive impact of social isolation.

